# THE EXAMINATION OF DETERMINANTS AND BARRIERS TO END-OF-LIFE DECISION MAKING AND PLANNING

**DOI:** 10.1093/geroni/igz038.882

**Published:** 2019-11-08

**Authors:** Brittany E Gaines, Debra J Dobbs

**Affiliations:** 1 University of Massachusetts Boston, Boston, Massachusetts, United States; 2 University of South Florida, Tampa, Florida, United States

## Abstract

As individuals are living longer, in many cases with chronic diseases, there is an increased focus on end-of-life (EOL) planning and decision making. This includes a broad spectrum of choices including advance care planning (ACP) and turning to palliative care or hospice care. Although there has been an increase in palliative and hospice care enrollment and ACP engagement over the past decade, participation remains low for certain subgroups of the population. The purpose of this symposium is to offer insight into reasons for these varying rates of engagement by exploring determinants and barriers to EOL decision making and planning and by examining caregiver knowledge of EOL decision making and planning from the service provider perspective. The first three studies examine various types of influences in EOL decision making and planning. Inoue and colleagues explore factors associated with the length of hospice stay, and Gaines and colleagues examine the impact of environmental characteristics in ACP. Ornstein and colleagues use Denmark registry data to assess the role of kinlessness at the time of death in EOL decision making and healthcare utilization. The final presentation by Noh and colleagues examines how service providers in rural areas perceive community residents’ knowledge of ACP and palliative care. The discussion following these presentations will compare findings across different forms of EOL decision making and planning, consider the impact of the varying methodological approaches used, and highlight implications of these works for potential interventions and policies related to EOL decision making and planning.

